# Age Structure, Growth, and Survival Rates of an Insular Population of *Hemidactylus turcicus*

**DOI:** 10.1002/ece3.71735

**Published:** 2025-07-03

**Authors:** Abdullah Altunışık, Didem Kurtul, Çiğdem Gül, Begüm Boran, Murat Tosunoğlu

**Affiliations:** ^1^ Biology Department, Faculty of Arts and Sciences Recep Tayyip Erdoğan University Rize Türkiye; ^2^ Department of Biology, School of Graduate Studies Çanakkale Onsekiz Mart University Çanakkale Türkiye; ^3^ Department of Biology, Faculty of Science Çanakkale Onsekiz Mart University Çanakkale Türkiye

**Keywords:** lifespan, Mediterranean gecko, Reptilia, sexual dimorphism, skeletochronology

## Abstract

The Mediterranean house gecko, *Hemidactylus turcicus* (Linnaeus, 1758), a widespread nocturnal lizard, exhibits diverse life‐history traits, yet its insular populations in Türkiye are underexplored. This study examines how Bozcaada's insular environment shapes the age structure, body size, growth rates, and sexual dimorphism of *H. turcicus* , providing a detailed demographic and morphometric baseline. We sampled 30 individuals (19 males, 11 females) from Bozcaada, Çanakkale, Türkiye, measuring snout‐vent length (SVL), body mass, and additional traits (head length, width, height, forearm, and hind leg length). Age was determined via skeletochronology, counting phalangeal Lines of Arrested Growth (LAGs). Results show a maximum longevity of 7 years for males (mean: 4.26 ± 0.33) and 6 years for females (mean: 3.91 ± 0.41). SVL averaged 45.19 ± 1.59 mm for males and 43.51 ± 2.33 mm for females, with growth rates of 3.10 ± 1.03 mm/year (males) and 3.58 ± 1.24 mm/year (females), modeled using the von Bertalanffy equation. Sexual dimorphism was subtle (SDI = 0.04, male‐biased), with no significant differences in morphometric traits between sexes. Survival rates were 0.78 for males and 0.76 for females, yielding adult life expectancies of 6.06 years (males) and 5.80 years (females), indicating a stable population. These findings suggest that Bozcaada's insular conditions, including limited resources and reduced predation, influence size, growth, and survival. This study establishes a novel profile of *H. turcicus* in an insular habitat, highlighting ecological adaptations and providing a foundation for future research and conservation strategies for this adaptable species.

## Graphical abstract

This study provides a comprehensive analysis of the life history traits of the Mediterranean house gecko ( *Hemidactylus turcicus* ) in the insular environment of Bozcaada, Türkiye, offering novel insights into its demography, morphometry, and ecological adaptations. The Mediterranean house gecko is a widespread and adaptable species, yet its insular populations remain underexplored, particularly in Türkiye. Our study addresses this gap by examining age structure, body size, growth rates, survival rates, and sexual dimorphism in the Bozcaada population using skeletochronology and the von Bertalanffy growth model.

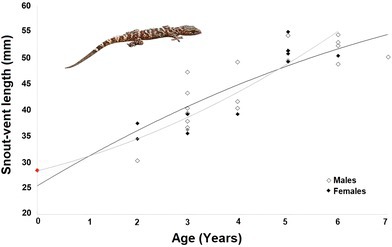

## 1 Introduction

Life‐history traits such as age structure, body size, growth rates, and sexual dimorphism are fundamental to understanding the ecology, evolutionary dynamics, and conservation needs of reptiles. These traits are shaped by a complex interplay of environmental factors, including habitat stability, resource availability, and predation pressure, which often differ significantly between mainland and island populations of the same species (Stearns 1992; Sinsch et al. 2007). Continental environments typically offer greater resource abundance and more stable conditions, supporting higher survival rates and less constrained growth (Adolph and Porter 1996). In contrast, islands impose unique ecological constraints—such as limited space, reduced predation, and variable resource availability—that can drive distinct adaptations, including shifts in body size, longevity, and reproductive strategies (Lomolino 2005; Altunışık et al. 2024; Ağdağ et al. 2025).

The Mediterranean house gecko, *Hemidactylus turcicus* (Linnaeus, 1758), is a small, nocturnal lizard widely distributed across the Mediterranean Basin, including southern Europe, northern Africa, the Middle East, and parts of Asia (Baran et al. 2012; Rato et al. 2011). In Türkiye, it thrives along the Mediterranean and Aegean coasts, with recent expansions into inland regions like Afyonkarahisar and Şanlıurfa, showcasing its ecological versatility (Cihan 2007; Uğurtaş et al. 2007). Its success in both natural habitats (e.g., rocky outcrops, forest edges) and human‐modified environments (e.g., settlements) is largely attributed to its adhesive toe pads, enabling it to exploit diverse substrates (Göçmen and Budak 2014; Locey and Stone 2006). Previous studies in Türkiye have documented regional variation in longevity—ranging from 5 to 9 years—and body size, with snout‐to‐vent length (SVL) differing across populations (Kanat and Tok 2015; Altunışık 2017; Göğebakan et al. 2024). Growth rates, often modeled with the von Bertalanffy equation, slow after sexual maturity—a trait typical of indeterminate growers—and are influenced by local conditions (Day and Taylor 1997; Altunışık 2017). Sexual dimorphism also varies subtly, from weak male bias in Adana (SDI = 0.027) to slight female bias in Muğla (Altunışık 2017; Kanat and Tok 2015). These differences are often linked to environmental factors such as temperature and resource availability, which profoundly influence growth and survival in ectotherms (Castanet 1994; Patnaik 1994; Ergul Kalaycı et al. 2015). Globally, males in the southeastern United States exhibit larger SVLs and heads, driven by sexual selection pressures like male–male competition (Granatosky and Krysko 2014; Saenz and Conner 1996), while female size may reflect reproductive investment (Blanckenhorn 2005; Vincent and Herrel 2007).

Despite these advances, detailed morphometric data (e.g., head length [HL], head width [HW], head height [HH], upper arm length [UAL], and hind leg length [HLL]) and demographic parameters such as growth rates, survival rates, and adult life expectancy remain undocumented for *H. turcicus* in Türkiye. Moreover, insular populations, such as those on Bozcaada in the Aegean Sea, are underexplored. Bozcaada's unique ecological context—characterized by a maritime climate, limited area, and reduced predation (Kurtul 2023)—may shape life history traits differently than mainland settings. We hypothesize that the insular environment of Bozcaada influences *H. turcicus* by constraining body size and growth rates while enhancing survival due to reduced ecological pressures, potentially altering patterns of sexual dimorphism compared to mainland populations. This study addresses the research question: How do age structure, body size, growth, and sexual dimorphism in the Bozcaada population of *H. turcicus* differ from mainland populations, and what novel insights do morphometric and demographic parameters provide? Our specific aim is to determine the age structure, SVL, growth rates, survival rates, adult life expectancy, and sexual dimorphism—including previously unreported morphometric characters (HL, HW, HH, UAL, and HLL)—of *H. turcicus* in Bozcaada using skeletochronology, providing a comprehensive baseline for comparison with mainland populations and advancing knowledge of its adaptability in insular habitats.

## 2 Materials and Methods

### 2.1 Species and Study Sites

*H. turcicus* is a nocturnal species known for its adaptability to diverse habitats. In Bozcaada, the gecko inhabits a variety of microenvironments, including human settlements (e.g., house walls), rocky terrains, and forest edges (Kurtul 2023, Figure 1). This adaptability aligns with previous observations of the species thriving in edificarian and natural settings across its range (Das et al. 2014). Bozcaada, located at 26°01′37″E and 39°49′29″N, experiences a Mediterranean climate characterized by hot, dry summers and mild, wet winters (Meteoblue 2025). Situated approximately 6 km from the Çanakkale Strait's Aegean exit, the island spans 36.03 km^2^ and is influenced by maritime conditions (Kurtul 2023). These climatic features—moderate temperatures and seasonal precipitation—support the nocturnal activity of *H. turcicus*, providing favorable conditions for foraging and reproduction. Bozcaada's topography includes low hills and coastal plains, with a maximum elevation not exceeding a few hundred meters (Kurtul 2023). The island's small size and proximity to the sea result in a relatively flat landscape interspersed with rocky outcrops and sparse vegetation. This terrain supports the gecko's climbing abilities, facilitated by its broad, adhesive toe pads, a trait typical of the genus *Hemidactylus* (Göçmen and Budak 2014).

**FIGURE 1:**
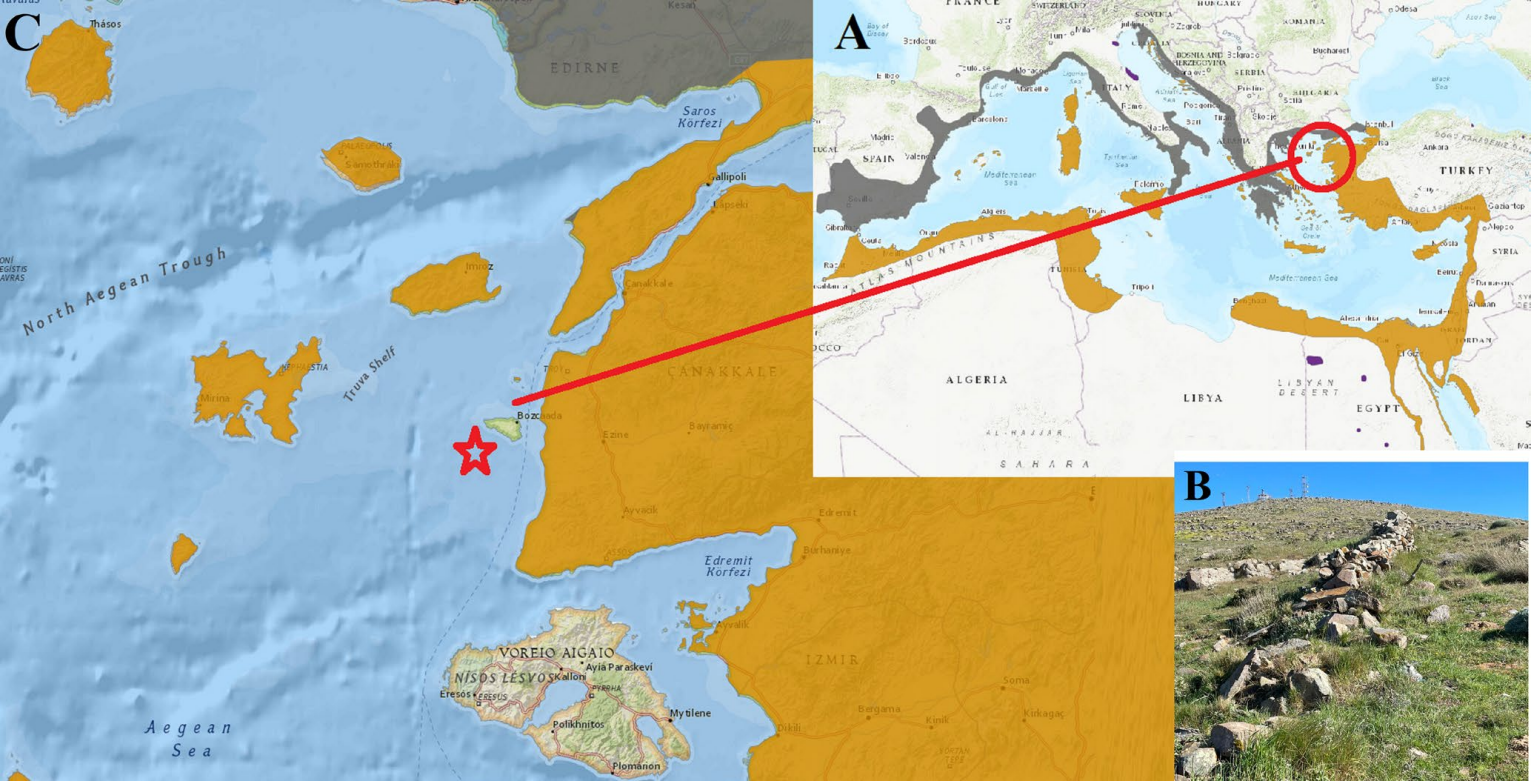
General distribution map of *H. turcicus* from IUCN (2025) (A), its habitat (B), and sampling locality in this study in Bozcaada, Aegean Sea (C).

A total of 30 (19 males, 11 females) Mediterranean house geckos, *H. turcicus* , were collected from Bozcaada, Türkiye, between March and August 2022. Gonads were examined for sex determination. Çanakkale 18 Mart University's Animal Experiments Ethics gave authorization (decision number: 2021/06‐14) for sampling.

A digital caliper (Mitutoyo, Japan) was employed to measure the SVL, total length (TL), HL, HW, HH, UAL, and HLL of the specimens. The specimens' body mass was measured to the nearest 0.01 g using balances. Subsequently, the toe from the right hind limb was excised and stored in a 70% ethanol solution, following the preservation guidelines outlined by Smirina (1994).

### 2.2 Age Assessment

Skeletochronology, a well‐established method for determining age distribution in numerous ectothermic species (Tatlı and Altunışık 2024; Altunışık et al. 2023), was utilized. This technique relies on the identification of “growth markers” or “Lines of Arrested Growth” (LAGs), visible in bone structures such as phalanges, femurs, tibias, and humeri. These LAGs form due to diminished metabolic activity in bone tissue during periods of dormancy or hibernation (Gibbons and McCarthy 1983).

**FIGURE 2:**
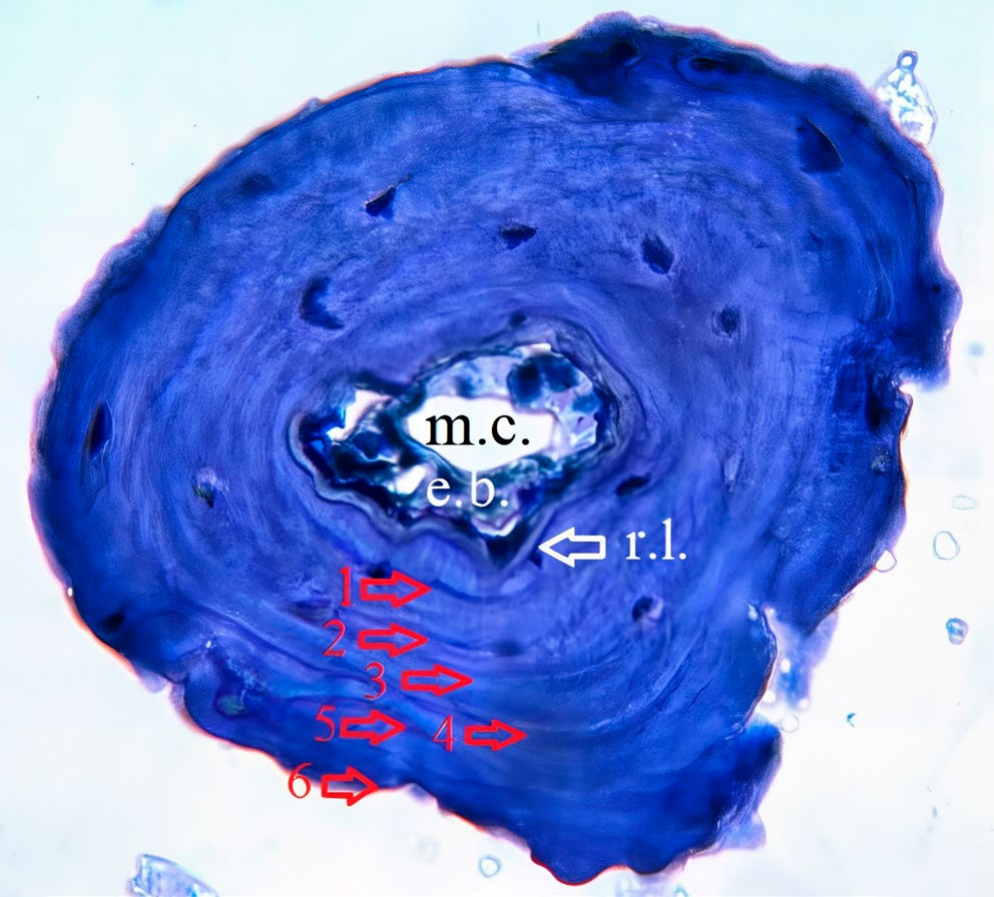
Cross‐sections (18 μm thick) of the phalangeal bone at the diaphysis level from *Hemidactylus turcicus* specimens. Arrows indicate Lines of Arrested Growth (LAGs). e.b., Endosteal bone; m.c., Marrow cavity; r.l., Resorption line.

The skeletochronological analysis was conducted using a modified approach based on Altunışık et al. (2022), while adhering to the protocols established by Smirina (1994). After preservation in 70% ethanol, the second phalanx was soaked in distilled water for 24 h and then decalcified in a 5% HNO₃ solution for approximately 2 h (Altunışık and Eksilmez 2018). Cross‐sections, 18 μm thick, were prepared using a Shandon Cryostat microtome. These sections were stained with Ehrlich's hematoxylin for 10 min. To mitigate the impact of endosteal resorption—which may obscure the innermost LAGs—sections with the smallest bone marrow cavities were selected and mounted in a glycerine solution. The prepared samples were observed and photographed under a light microscope with a Pixera digital camera at ×10 and ×20 magnifications (Figure 2). The LAGs were independently counted and cross‐verified by the researchers after a detailed evaluation of all images (Altunışık and Eksilmez 2021; Ağdağ et al. 2025).

### 2.3 Statistical Analysis and Software

Statistical analysis of the experimental data was performed using SPSS v. 29.0 (IBM Corp.), with *α* = 0.05. Data normality was assessed with the Shapiro–Wilk test, followed by the application of either parametric or nonparametric (e.g., Mann–Whitney *U*) tests to compare differences between sexes or populations. Multivariate differences among morphometric traits were analyzed by MANOVA, and ANCOVA was used to compare body mass while controlling for SVL as a covariate. The association between SVL and age was examined using Pearson's correlation coefficient. Sexual dimorphism was quantified using the Sexual Dimorphism Index (SDI), as proposed by Lovich and Gibbons (1992):

(1) $$\mathrm{SDI}=\left(\left(\text{size of larger}\;\mathrm{sex}\right)/\left(\text{size of smaller}\;\mathrm{sex}\right)\right)–1$$

Survival rates (Svr) were calculated based on the method described by Robson and Chapman (1961):

(2) $$\mathrm{Svr}=T/\left(R+T–1\right)$$

Here, *T* = *n*₁ + 2*n*₂ + 3*n*₃ + 4*n*₄ + …, *R* = Σ*nₛ*, and *nₛ* represents the number of individuals in each age class. This approach assumes a stable annual survival rate. The distance between consecutive LAGs served as a dependable measure of annual individual growth. A notable reduction in the spacing between two adjacent LAGs was interpreted as the onset of sexual maturity (Ryser 1988). Adult lifespan (ESP), defined as the expected longevity of individuals reaching sexual maturity, was estimated using Seber's (1973) formula:

(3) $$\mathrm{ESP}=0.5+1/\left(1–\mathrm{Svr}\right)$$

Growth patterns were modeled using the von Bertalanffy growth equation, consistent with prior studies (Guarino et al. 2010; Kara et al. 2025):

(4) $${\mathrm{SVL}}_{\left(c\right)}={\mathrm{SVL}}_{\left(\max\right)}\;\left(1-e^{-k\left(c-t_{0}\right)}\right)$$

In this equation, SVL_(c)_ is the size at age c, SVL_(max)_ is the maximum asymptotic SVL, *e* is Euler's number (2.718), *k* is the growth rate coefficient, and *t*₀ is the age at metamorphosis.

MS Excel was used to compute SVL_(max)_, *k*, and growth rates, with the latter derived from the equation *r* = *k* (SVL_(max)_ – SVL_(*t*)_). Variations in growth rates within and across populations were analyzed using *t‐*tests.

## 3 Results

**FIGURE 3:**
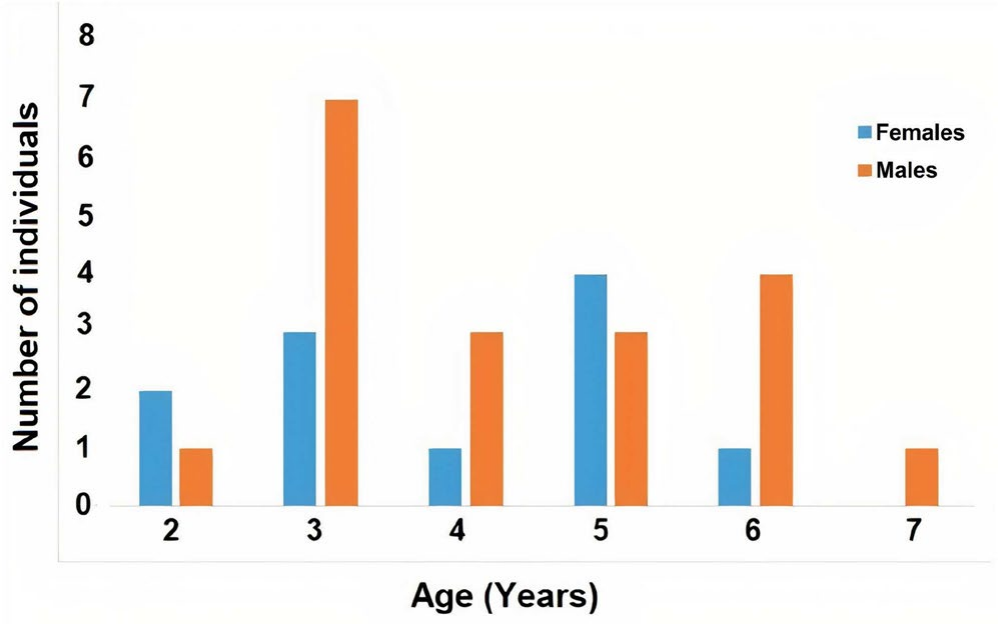
Age distribution in Bozcaada populations of *Hemidactylus turcicus* .

Adult individuals in the Bozcaada population had ages spanning 2–7 years for males (mean: 4.26 ± 0.33) and 2–6 years for females (mean: 3.91 ± 0.41) (Table 1). Statistical analysis showed no significant difference in mean age between sexes (Mann–Whitney *U* Test; *U* = 90.00, df = 28, *p* = 0.52). Examination of phalangeal cross‐sections indicated that 60% of adults displayed endosteal resorption, resulting in partial degradation of periosteal bone near the medullary cavity's edge (Figure 2). The predominant age class was the third year, comprising 10 individuals (34%), followed by the fifth year with seven individuals (23%) (Figure 3). Both males and females attained sexual maturity between 2 and 3 years. The anticipated postmaturity lifespan (ESP) for individuals reaching this stage was estimated at 6.06 years for males and 5.80 years for females (Table 2). Annual survival probabilities (Svr) were 0.78 for males and 0.76 for females, translating to 78% and 76% year‐to‐year survival, respectively (Table 2). Growth rates showed similarity between sexes, averaging 3.10 ± 1.03 mm/year for males and 3.58 ± 1.24 mm/year for females (*U*‐test, *U* = 12, *p* = 0.917).

**TABLE 1:** Descriptive statistics of age, body mass, and some morphometric traits in the studied population of *Hemidactylus turcicus* adults from Bozcaada island.

Sex	Traits	*N*	Minimum	Maximum	Mean	SE
	Age (years)	19	2	7	4.26	0.33
Males	Body mass (g)	19	0.60	3.50	2.15	0.19
	TOTL (mm)	14	61.62	207.55	95.13	9.11
	SVL (mm)	19	30.27	54.34	45.19	1.59
	HL (mm)	19	8.01	14.63	11.91	0.39
	HW (mm)	19	5.75	10.17	8.49	0.28
	HH (mm)	19	3.35	6.32	4.88	0.18
	UAL (mm)	19	8.84	15.95	12.73	0.50
	HLL (mm)	19	11.05	23.92	16.80	0.72
	Age (years)	11	2	6	3.91	0.41
Females	Body mass (g)	11	1.02	3.11	1.97	0.25
	TOTL (mm)	7	73.21	103.76	83.00	4.74
	SVL (mm)	11	34.51	54.95	43.51	2.33
	HL (mm)	11	8.36	14.09	11.33	0.57
	HW (mm)	11	6.15	10.66	8.54	0.49
	HH (mm)	11	3.43	6.51	4.58	0.30
	UAL (mm)	11	8.66	14.05	11.74	0.66
	HLL (mm)	11	9.69	20.31	15.07	0.91

Abbreviations: *N*, number of specimens; SE, standard error of the mean.

**TABLE 2:** Descriptive statistics of growth rate (mm per year), growth coefficient (*k*), ESP and Sr. in the studied population of *Hemidactylus turcicus* adults from Bozcaada island, Türkiye.

Sex	*N*	Growth rate ± SE	*k*	SVLmax	ESP	Svr	SDI
Males	19	3.10 ± 1.03	0.29	55.17	4.94	0.78	0.04
Females	11	3.58 ± 1.24	0.37	52.49	4.70	0.76	

Abbreviations: ESP, adult life expectancy; *N*, number of specimens; SDI, sexual dimorphism index; SE, standard error of the mean; Svr, survival rate.

In the Bozcaada population, male SVL ranged from 30.27 to 54.34 mm (mean: 45.19 ± 1.59 mm), while female SVL ranged from 34.51 to 54.95 mm (mean: 43.51 ± 2.33 mm).

**FIGURE 4:**
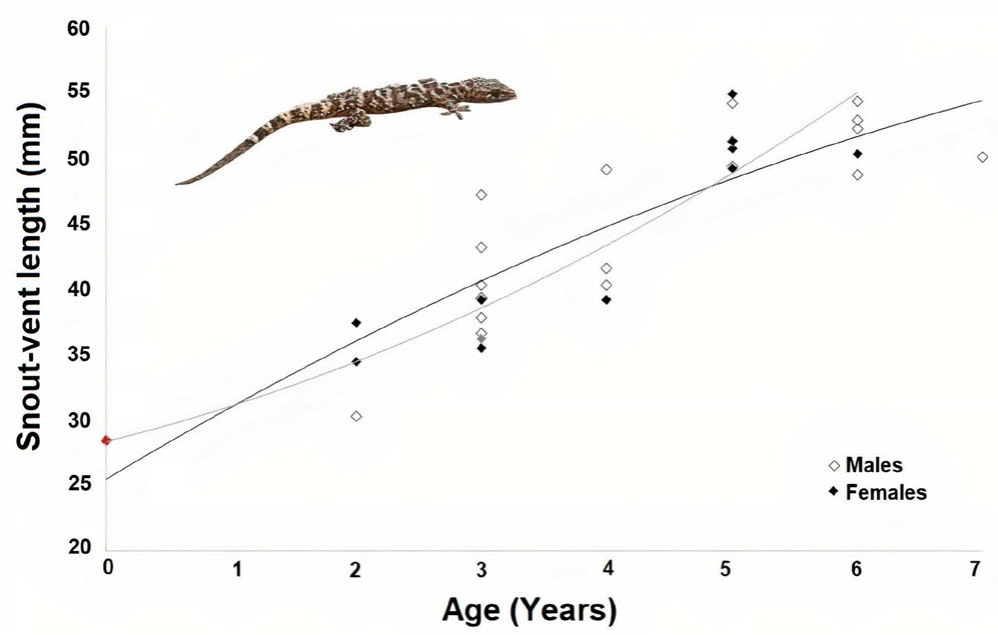
The von Bertalanffy growth model concerning SVL and age in the Bozcaada population of *Hemidactylus turcicus* .

Growth parameters derived from the von Bertalanffy model effectively described the relationship between SVL and age (Figure 4).

No notable sex‐based difference in SVL was detected (MANOVA, *F* = 0.376, df = 1, *p* = 0.545). The SDI was computed as 0.04, reflecting slight dimorphism. The maximum observed SVL for females exceeded the asymptotic value (SVLmax: females, 52.49 mm; males, 55.17 mm; Table 2).

Male body mass averaged 2.16 ± 0.19 g (range: 0.60–3.50 g), while female body mass averaged 1.97 ± 0.26 g (range: 1.02–3.11 g). Statistical tests indicated no significant sex difference in body mass (MANOVA, *F* = 0.379, df = 1, *p* = 0.543), even when SVL was accounted for (ANCOVA, *F* = 0.09, df = 1, *p* = 0.92). Similarly, morphometric features including HL, width, height, forearm length, and HLL no significant variation between males and females (MANOVA, *p* > 0.05). Within this population, strong positive relationships were observed between SVL and age (Pearson's *r* = 0.86, *p* < 0.001), SVL and body mass (*r* = 0.95, *p* < 0.001), and age and body mass (*r* = 0.806, *p* < 0.001).

## 4 Discussion

This study aimed to elucidate how the insular environment of Bozcaada shapes the life‐history traits of *He. turcicus* , particularly in comparison to mainland populations, by examining age structure, body size, growth rates, and sexual dimorphism. The Bozcaada population exhibits a maximum longevity of 7 years for males and 6 years for females, with mean ages of 4.26 ± 0.33 and 3.91 ± 0.41 years, respectively, aligning closely with the 7‐year maximum reported in Adana (Altunışık 2017). However, it falls short of the 8–9 years observed in Muğla (Kanat and Tok 2015) and exceeds the 5 years in İzmir (Göğebakan et al. 2024). These differences likely reflect environmental influences on age structure, a well‐documented phenomenon in ectotherms (Castanet 1994; Patnaik 1994). Bozcaada's maritime climate, with mild winters and moderate temperatures (Kurtul 2023), may support a lifespan intermediate between the warmer, resource‐rich conditions of Muğla and the potentially more stressful urban setting of İzmir (Göğebakan et al. 2024). Compared to other geckos, such as *H. brooki* (4 years; Pancharatna and Kumbar 2005) or *Mediodactylus kotschyi* (8 years; Çiçek et al. 2015), *H. turcicus* in Bozcaada shows a moderate lifespan, suggesting a balanced trade‐off between growth and survival in its insular niche.

Body size in Bozcaada (males: 45.19 ± 1.59 mm; females: 43.51 ± 2.33 mm) is smaller than in Muğla (males: 50.53 mm (*n* = 12); females: 51.19 mm (*n* = 18); Kanat and Tok 2015) and Adana (males: 50.69 mm (*n* = 9); females: 49.35 mm (*n* = 10); Altunışık 2017), but comparable to İzmir (males: 44.4 mm (*n* = 11); females: 48.7 mm (*n* = 18); Göğebakan et al. 2024). The von Bertalanffy growth model indicates asymptotic SVLs (males: 55.17 mm; females: 52.49 mm) similar to mainland populations, yet the observed SVLs suggest Bozcaada geckos may not reach their full potential size, possibly due to limited resources or habitat constraints on the island (Kurtul 2023). Growth rates (males: 3.10 ± 1.03 mm/year; females: 3.58 ± 1.24 mm/year) are consistent with the slowing postmaturity growth typical of lizards (Day and Taylor 1997), and the strong SVL‐age correlation (*r* = 0.86, *p* < 0.001) mirrors findings in Muğla (*r* = 0.96–0.98; Kanat and Tok 2015) and İzmir (*r* = 0.87; Göğebakan et al. 2024). This pattern aligns with global observations of *H. turcicus* , where body size correlates positively with age but plateaus after sexual maturity (Granatosky and Krysko 2014).

Sexual maturity at 2–3 years in Bozcaada is consistent across Turkish populations (Altunışık 2017; Kanat and Tok 2015; Göğebakan et al. 2024) and corresponds to an SVL of approximately 44 mm, as noted by Selcer (1986). This stability suggests a conserved life history trait, likely tied to a size threshold for reproductive viability (Adolph and Porter 1996). However, the subtle sexual dimorphism (SDI = 0.04, male‐biased) contrasts with variable patterns elsewhere: Adana shows a weak male bias (SDI = 0.027; Altunışık 2017), Muğla a slight female bias (Kanat and Tok 2015), and İzmir no significant difference (Göğebakan et al. 2024). In the southeastern United States, males exhibit larger SVLs and heads, linked to sexual selection (Granatosky and Krysko 2014; Saenz and Conner 1996), whereas Bozcaada's minimal dimorphism may reflect reduced competition or resource limitation on the island (Blanckenhorn 2005; Vincent and Herrel 2007). The lack of significant differences in morphometric traits (e.g., head size, limb length) further supports this, differing from studies where male head size drives dimorphism (Saenz and Conner 1996).

Survival rates (Svr: males 0.78; females 0.76) and expected postmaturity lifespans (ESP: males 6.06 years; females 5.80 years) indicate a stable population with moderate annual mortality, higher than implied by İzmir's shorter lifespan (Göğebakan et al. 2024) but lower than Muğla's potential (Kanat and Tok 2015). Bozcaada's mild climate and prey availability (Kurtul 2023) likely bolster survival, though its small size may cap population expansion (Locey and Stone 2006). Endosteal resorption in 60% of adults, higher than in Adana (Altunışık 2017), aligns with patterns in older individuals (Göğebakan et al. 2024), necessitating careful section selection to avoid age underestimation (Castanet et al. 1993).

In conclusion, the Bozcaada population exhibits intermediate longevity, smaller body sizes, and minimal sexual dimorphism compared to mainland *H. turcicus* populations, reflecting the influence of its insular environment. These traits suggest adaptations to a constrained yet stable habitat, consistent with broader patterns in ectotherm life history (Castanet 1994; Shine 1989). Further research comparing insular and mainland populations could clarify how climate, isolation, and ecological pressures shape *H. turcicus* demography, enhancing conservation strategies for this adaptable species.

## 5 Conclusion

The Bozcaada population of *H. turcicus* reveals distinct life‐history traits shaped by its insular environment, characterized by a maximum longevity of 7 years for males and 6 years for females, smaller body sizes (males: 45.19 ± 1.59 mm; females: 43.51 ± 2.33 mm), and subtle male‐biased sexual dimorphism (SDI = 0.04). These traits position Bozcaada geckos as intermediate in longevity compared to mainland populations like Muğla (8–9 years) and İzmir (5 years), while their smaller sizes and moderate growth rates (males: 3.10 ± 1.03 mm/year; females: 3.58 ± 1.24 mm/year) suggest resource or habitat constraints unique to the island. Survival rates (males: 0.78; females: 0.76) and expected adult lifespans (males: 6.06 years; females: 5.80 years) indicate a stable population, likely supported by Bozcaada's mild maritime climate and reduced predation pressure. Minimal sexual selection pressures are further supported by the lack of significant morphometric differences in head size and limb length, contrasting with patterns observed in some mainland and global populations. These findings align with our hypothesis that insular conditions constrain growth and size while enhancing survival, reflecting adaptive trade‐offs in this ecologically versatile species. By providing the first detailed demographic and morphometric baseline for an insular Turkish population, this study enhances our understanding of *H. turcicus* adaptability. Future research should explore additional insular populations and incorporate genetic analyses to disentangle environmental and evolutionary drivers, informing conservation strategies for this widespread gecko in diverse habitats.

## Author Contributions

**Abdullah Altunışık:** formal analysis (equal), investigation (equal), methodology (equal), supervision (equal), visualization (equal), writing – review and editing (equal). **Didem Kurtul:** conceptualization (equal), data curation (equal), project administration (equal), resources (equal), writing – review and editing (equal). **Çiğdem Gül:** conceptualization (equal), formal analysis (equal), investigation (equal), writing – review and editing (equal). **Begüm Boran:** conceptualization (equal), data curation (equal), project administration (equal), resources (equal). **Murat Tosunoğlu:** conceptualization (equal), supervision (equal), writing – review and editing (equal).

## Conflicts of Interest

The authors declare no conflicts of interest.

## Data Availability

All data generated or analyzed during this study are included in this manuscript.
